# Multi-Modal CLIP-Informed Protein Editing

**DOI:** 10.34133/hds.0211

**Published:** 2024-12-19

**Authors:** Mingze Yin, Hanjing Zhou, Yiheng Zhu, Miao Lin, Yixuan Wu, Jialu Wu, Hongxia Xu, Chang-Yu Hsieh, Tingjun Hou, Jintai Chen, Jian Wu

**Affiliations:** ^1^ School of Medicine, Zhejiang University, Hangzhou, China.; ^2^ College of Computer Science and Technology, Zhejiang University, Hangzhou, China.; ^3^ Medical Big Data Center, Guangdong Provincial People’s Hospital (Guangdong Academy of Medical Sciences), Southern Medical University, Guangzhou, China.; ^4^ Innovation Institute for Artificial Intelligence in Medicine of Zhejiang University, College of Pharmaceutical Sciences, Zhejiang University, Hangzhou, China.; ^5^ AI Thrust, Information Hub, HKUST (Guangzhou), Guangzhou, China.; ^6^ Second Affiliated Hospital School of Medicine, Hangzhou, China.; ^7^ School of Public Health, Zhejiang University, Hangzhou, China.; ^8^ Institute of Wenzhou, Wenzhou, China.

## Abstract

**Background:** Proteins govern most biological functions essential for life, and achieving controllable protein editing has made great advances in probing natural systems, creating therapeutic conjugates, and generating novel protein constructs. Recently, machine learning-assisted protein editing (MLPE) has shown promise in accelerating optimization cycles and reducing experimental workloads. However, current methods struggle with the vast combinatorial space of potential protein edits and cannot explicitly conduct protein editing using biotext instructions, limiting their interactivity with human feedback. **Methods:** To fill these gaps, we propose a novel method called ProtET for efficient CLIP-informed protein editing through multi-modality learning. Our approach comprises 2 stages: In the pretraining stage, contrastive learning aligns protein–biotext representations encoded by 2 large language models (LLMs). Subsequently, during the protein editing stage, the fused features from editing instruction texts and original protein sequences serve as the final editing condition for generating target protein sequences. **Results:** Comprehensive experiments demonstrated the superiority of ProtET in editing proteins to enhance human-expected functionality across multiple attribute domains, including enzyme catalytic activity, protein stability, and antibody-specific binding ability. ProtET improves the state-of-the-art results by a large margin, leading to substantial stability improvements of 16.67% and 16.90%. **Conclusions:** This capability positions ProtET to advance real-world artificial protein editing, potentially addressing unmet academic, industrial, and clinical needs.

## Introduction

Proteins are vital components of biological systems, executing a myriad of functions that underpin an extensive array of cellular processes and biological pathways [1]. Throughout billions of years of evolution, proteins undergo changes in their sequences and structures, which further influence their functional properties. Protein editing, or protein modification, is a natural process that gradually increases the diversity of protein structures and functions over time, offering valuable insights into the controlled discovery and optimization of proteins.

Artificial protein editing approaches, which emulate natural evolutionary processes, have made remarkable strides and are now widely applied in healthcare, serving as valuable tools for probing natural systems, creating therapeutic conjugates, and generating novel protein constructs. In cancer vaccine development, they enable precise modifications of protein sequences or structures, leading to the creation of more effective and less toxic drugs tailored to an individual’s genome [2–5], or enhancing drug delivery to tumor cells while minimizing side effects on healthy tissues [6,7]. In gene therapy, protein editing approaches are extensively used to harness programmable nucleases, allowing for precise cutting and pasting of genetic information in living cells and organisms [8–10]. Additionally, protein editing helps reduce the emergence of drug resistance [11,12] by modifying proteins involved in resistance pathways, thereby restoring sensitivity to existing therapies and enabling the development of novel, more potent treatments [13,14]. This marks further progress in the field of precision medicine [15]. Although there have been some notable achievements, the challenge of protein editing lies in its controllability. Achieving controllable protein editing is difficult because the space of possible proteins is vastly larger than the subset with desired functions. Moreover, organizing human-understandable “communications” to control proteins and facilitate “human–protein” interactions remains a substantial obstacle.

In the past 2 decades, various methodologies have been developed for posttranslational protein modifications (PTMs), aiming to artificially edit amino acids to enhance human-expected properties. When applied in a biologically benign manner, these methodologies have the potential to form the foundation of true synthetic biology [16], but still heavily rely on time-consuming and post hoc wet laboratory engineering [5]. Recently, machine learning-based methods have demonstrated great promise in a wide range of protein-related applications, including 3-dimensional (3D) structure prediction [17,18], mutation effects prediction [19], functionality prediction [20–22], and de novo protein design [23,24]. With the development of large language models (LLMs), protein language models (PLMs) pretrained on large-scale protein sequence corpora have succeeded in acquiring powerful protein representations, showcasing outstanding performance across diverse tasks [25–27]. Researchers have also developed machine learning-assisted protein editing (MLPE) approaches, allowing in silico searching for all edited candidates and potentially improving wet-lab protein editing performance. Although these deep learning-based methods have effectively learned and identified protein expression spaces, partially improving controllability, most existing approaches still heavily rely on black-box optimization algorithms, which iteratively sample edited proteins and use fitness predictors, trained on selected informative samples, to guide the editing direction. The iterative refinement within the vast combinatorial space of edited protein sequences still heavily constrains the performance and efficiency of MLPE approaches. Recently, multi-modality learning framework like CLIP [28] has shown promising results in image–text retrieval [29–33], CLIP-informed image classification [31,33–35], natural language visual reasoning [29,36] and text-guided image editing [37–39], illuminating an exciting opportunity on protein editing.

*Can we efficiently control protein editing under the guidance of biological texts with large PLMs?*

This paper proposes a generic protein editing method named ProtET. ProtET is a multi-modal approach that can accomplish proximally constrained protein editing toward desired properties. Inspired by CLIP [28], which establishes a mapping between image feature space and natural language feature space to enable language-based control of image space, ProtET learns cross-modality mapping between proteins and natural languages to achieve controllable protein editing. ProtET proposes an innovative hierarchical training paradigm, incorporating a multi-modal pretraining phase and a cross-modal generation phase. Compared to existing machine learning-based protein editing methods (e.g., Single-Mutant [40], AFP-DE [41], and EvoPlay [4]), ProtET achieves promising performance on the established protein editing tasks, demonstrating substantial functionality improvements across multiple attribute domains including enzyme catalytic activity, protein stability, and antibody-specific binding ability. This highlights ProtET as a valuable tool for future controllable protein discovery and optimization endeavors in real-world scenarios.

## Methods

### Overview of ProtET

**Fig. 1.**
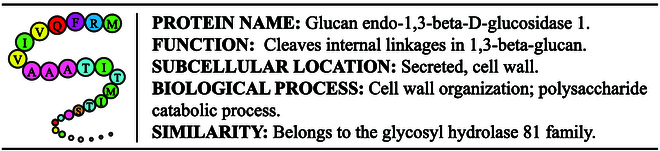
An illustration of the protein–biotext pair. The textual descriptions include the protein’s name, function, subcellular location, biological process, and similarity to other proteins.

ProtET is a multi-modality deep learning model that hybridly encodes biological languages and natural languages, and then executes cross-modal generation to achieve controllable protein editing. To accomplish this, we first curate millions of protein–biotext aligned pairs, each comprising protein sequences and functional biotext annotations, as illustrated in Fig. 1. The large-scale multi-modal dataset consists of 570,420 proteins with manually reviewed property annotations and 251,131,639 proteins with computationally analyzed annotations. We then construct transformer-structured encoder-based models (i.e., a large protein model with 650 million trainable parameters and an LLM with 100 million trainable parameters) to encode the features of both protein sequences and biotexts, respectively. Additionally, a hierarchical training paradigm is proposed to alleviate the challenge of cross-modal protein editing. During the pretraining stage, similar to CLIP [28], our multi-modality pretraining is performed using contrastive learning objectives to align the features of the protein and biotext, facilitating easier editing instruction. In the editing stage, the aligned protein features and desired function description features extracted by the pretrained models are fused by the introduced FiLM module. We construct a generative decoder model to design the desired protein sequences in an autoregressive manner. ProtET innovatively introduces a novel protein editing paradigm through multi-modal pretraining and cross-modal generation. Its controllable protein editing capability to enhance human-expected functionality demonstrates the great potential for clinical applications, such as vaccine development and genetic therapy.

### Curated protein–biotext dataset

**Fig. 2.**
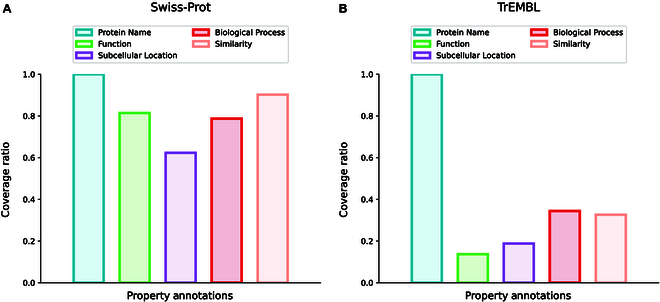
Coverage ratios of protein property annotations in (A) Swiss-Prot and (B) TrEMBL.

To attain multi-modal protein–biotext pretraining, we first build a protein–biotext paired dataset. Swiss-Prot database strives to provide a high level of manually reviewed protein annotations, with a minimal level of redundancy [42]. TrEMBL consists of protein entries with computationally analyzed annotations, derived from the translation of EMBL (European Molecular Biology Laboratory) nucleotide sequences [42]. Given protein database with rich, consistent, and accurate protein functional annotations, we curate a new multi-modal dataset with aligned pairs of protein sequences and biotext functional annotations [43]. Concretely, proteins with elaborate annotations are downloaded from Swiss-Prot and TrEMBL in January 2024, yielding 570,420 and 251,131,639 sequences, respectively. For protein sequences collected from Swiss-Prot and TrEMBL, we select 5 property fields: (a) “Protein Name” gives the full protein name recommended by the UniProt consortium; (b) “Function” depicts diverse functions owned by a protein; (c) “Subcellular Location” describes the location and topology of a mature protein in the cell; (d) “Biological Process” represents larger processes accomplished by multiple molecular activities; (e) “Similarity” provides information about the protein families that a protein belongs to. The above biotext descriptions and the corresponding protein sequences are aligned in detail, as shown in Fig. 1. Additionally, we record the annotation coverage ratio of the aforementioned 5 selected property fields, as displayed in Fig. 2. And we present the detailed protein sequence amount at different evidence levels in Table 1. To further improve the quality of curated multi-modal dataset, we keep all manually reviewed protein annotations from Swiss-Prot and meticulously filter computationally analyzed protein annotations from TrEMBL. Specifically, we remove the protein entries having annotation coverage ratio less than 40%, as well as those with low evidence level at 4 and 5, resulting in 67,972,109 protein–biotext aligned pairs tailored for multi-modality pretraining.

**Table 1.** Protein sequence statistics of different evidence level in Swiss-Prot and TrEMBL

Evidence level ↓	“Protein Name”	“Function”	“Subcellular Location”	“Biological Process”	“Similarity”
Swiss-Prot					
1: Evidence at protein level	113,077	100,669	85,666	92,319	90,599
2: Evidence at transcript level	55,883	45,001	44,158	41,718	45,961
3: Inferred from homology	386,595	317,164	222,312	312,668	376,527
4: Predicted	13,032	1,600	3,048	2,282	810
5: Uncertain	1,833	303	732	483	728
TrEMBL					
1: Evidence at protein level	230,045	24,700	57,303	113,846	96,717
2: Evidence at transcript level	1,373,855	265,605	417,404	637,893	687,615
3: Inferred from homology	81,928,782	32,589,671	35,259,878	54,799,521	81,090,429
4: Predicted	167,598,957	1,573,895	11,568,640	30,878,461	31,248
5: Uncertain	0	0	0	0	0

### Multi-modality pretraining

Aiming to pave the way for CLIP-informed protein editing, we first need to extract features of the protein and biotext, and align feature spaces of both modalities. In this paper, we exploit 2 transformer-encoder-based LLMs to extract protein and biotext features, respectively. ESM-2 is employed as the protein sequence encoder, which is pretrained on millions of protein sequences [27]. In recent studies, ESM-2 has proven quite beneficial in many protein-related studies [44,45]. PubMedBERT is employed as the biotext description encoder, which is pretrained on PubMed article context [46]. And PubMedBERT is a robust biomedical language model customized to encode biomedical texts.

**Fig. 3.**
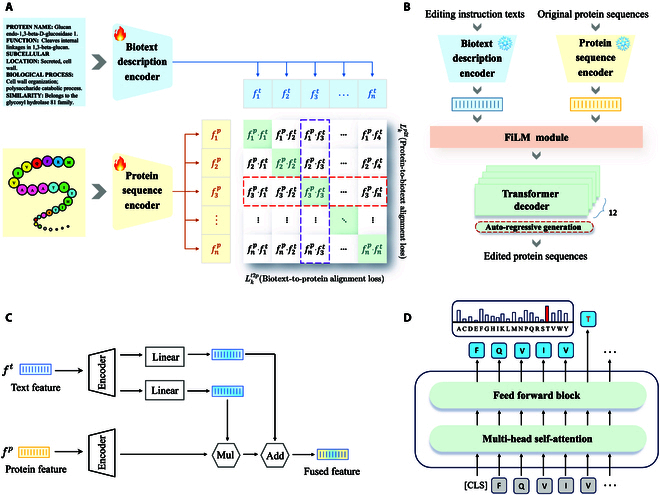
The workflow and framework details of ProtET. (A) A CLIP-like contrastive pretraining aligns features of protein sequences and biotext descriptions. (B) FiLM module and transformer-decoders for protein editing. The FiLM module integrates multi-modal features from the original protein sequences and the editing instruction texts, serving as the editing condition. Based on this condition, transformer-decoders design edited protein sequences through an autoregressive generation process. (C) Details of the FiLM module. It extracts multiplicative and additive factors from text features using linear mappings. These factors conditionally optimize protein features through addition and multiplication to create fused features. (D) Details of the transformer decoder. It uses a multi-head self-attention module to learn comprehensive residue–residue interactions and predicts the next residue based on the previous ones.

Inspired by extraordinary endeavors of image–text multi-modality alignment, we propose to align feature spaces of both modalities via contrastive learning [28,37,38,47]. As illustrated in Fig. 3A, in a list of protein–biotext pairs, $p_{i}$ and $t_{j}$ represent the $i_{\mathrm{th}}$ protein sequence and $j_{\mathrm{th}}$ biotext description, respectively. When i=j, $\left(p_{i},\;t_{j}\right)$ is a positive pair, and when i≠j, it is a negative pair. $f_{i}^{p}$ and $f_{i}^{t}$ are protein and biotext representations extracted by the aforementioned encoders, both projected to the same feature dimension. Following CLIP [28], given a batch of N protein–biotext pairs, there are N×N possible pairings including N correct pairings and $N^{2}-N$ incorrect pairings. The CLIP-informed model aims to learn a multi-modal aligned feature space via contrastive learning. Within a batch, the model maximizes the similarity scores of the protein and biotext embeddings from *N* correct pairings, and minimizes the similarity scores of the embeddings from $N^{2}-N$ incorrect pairings. We optimize a symmetric cross-entropy loss over these predicted pairing similarity scores. Formally, we introduce our contrastive learning objective, training our model to discriminate positive and negative protein–biotext pairs. The overall training objective is constructed of the protein-to-biotext alignment loss and biotext-to-protein alignment loss:

$$L_{i}^{p2t}=-\frac{1}{N}\text{log}\frac{\text{exp}\left(\mathrm{sim}\left(f_{i}^{p},\;f_{i}^{t}\right)/\tau \right)}{\sum_{j}\text{exp}\left(\mathrm{sim}\left(f_{i}^{p},\;f_{j}^{t}\right)/\tau \right)},$$ (1)

$$L_{i}^{t2p}=-\frac{1}{N}\text{log}\frac{\text{exp}\left(\mathrm{sim}\left(f_{i}^{t},\;f_{i}^{p}\right)/\tau \right)}{\sum_{j}\text{exp}\left(\mathrm{sim}\left(f_{i}^{t},\;f_{j}^{p}\right)/\tau \right)},$$ (2)

where $\mathrm{sim}\left(\;\cdot ,\cdot \right)$ denotes the similarity function (i.e., vector dot product in this paper) and τ denotes the temperature parameter that controls the softmax distribution.

Overall, the final contrastive learning objective in the multi-modality pretraining stage is formulated as:

$$L_{\text{align}}=\frac{1}{2}\sum_{i=1}^{N}\left(L_{i}^{p2t}+L_{i}^{t2p}\right).$$ (3)

where $L_{i}^{p2t}\;\text{and}\;L_{i}^{t2p}$ respectively represent the protein-to-biotext alignment and biotext-to-protein alignment loss.

### Protein editing generator

Owing to the aligned feature spaces accomplished by multi-modality pretraining introduced in the previous section, now we could encode the features of original protein sequences and editing instruction texts, and then construct a decoder to generate the edited protein sequences. As shown in Fig. 3B, the protein editing generator is composed of a feature fusion module and a generative decoder. Concretely, FiLM module [48] is leveraged to fuse multi-modal features from original protein sequences and editing instruction texts. Such architecture design aims to optimize protein features based on editing instruction text features, integrating multi-modal information to accomplish cross-modal protein editing. The generative decoder is intentionally constructed using 12 layers of transformer decoders, tailored for autoregressively generating edited protein sequences. Given fused features as the final editing condition, edited protein sequences are generated incrementally, one amino acid at a time, with each amino acid being conditioned on the probability of previously generated ones.

$$P\left(S_{\text{edited}}\right)=\prod_{i=1}^{n}P\left(s_{i}|s_{1},\;s_{2},\ldots ,\;s_{i-1};\;\text{FiLM}\left(S_{\text{original}},T_{\text{instruction}}\right)\right).$$ (4)

where $S_{\text{edited}}=\left(s_{1},\;s_{2},\ldots ,\;s_{n}\right)$ represents the edited protein sequences and $\left(S_{\text{original}},T_{\text{instruction}}\right)$ represents encoded features of original protein sequences and editing instruction texts, respectively.

Given the paired original and edited protein sequences, the protein editing generator is trained in an unsupervised manner to generate proteins with higher feature similarity to the editing instruction texts, aligning with human-expected functional attributes.

$$\begin{array}{c} \begin{array}{l} L_{\text{edit}}=H\left[\mathrm{sim}\left(S_{\text{original}},T_{\text{instruction}}\right)-\mathrm{sim}\left(S_{\text{edited}},T_{\text{instruction}}\right)\right]\\ \;+\text{SoftCrossEntropy}\left[y\left(s_{i}/S_{\text{original}}\right),\;p\left(s_{i}/S_{\text{edited}}\right)\right]. \end{array} \end{array}$$ (5)

where $s_{i}/S_{\text{original}}$ and $s_{i}/S_{\text{edited}}$ respectively denote the amino acid in the $i_{th}$ position of original protein sequences and edited protein sequences. $\mathrm{sim}\left(\;\cdot ,\cdot \right)$ denotes the cosine similarity function [49] and $H\left(\alpha \right)=\max\left(0,\;\alpha \right)$. The second item in $L_{\text{edit}}$ serves as a regularization term to avoid model collapse and guides the model to perform slight amino acid modification, gradually improving functional attributes.

### Implementation details

Following previous works [44,45,50], we use the base version of ESM-2 model with 650 million trainable parameters to encode protein sequences [27]. As for biotext encoding, we apply PubMedBERT with 12 transformer layers and 100 million trainable parameters. All protein sequences are padded or truncated to a fixed length of 1,024 tokens, and we align all the biotext descriptions to a unified length of 512. The embedding dimensions of protein and biotext LLMs are 1,280 and 768, respectively. Aiming to align multi-modal representations, we choose 512 to be the projected common feature dimension. The temperature coefficient τ is set as 0.01. For the curated multi-modal dataset, we use protein–biotext pairs from filtered TrEMBL for the pretraining stage, and those from Swiss-Prot are exploited to train protein editing generator. The overall framework is trained with a batch size of 128 for 10 epochs, utilizing 16 NVIDIA 32G V100 GPUs. The learning rate is initialized as $5.0\times 10^{-5}$ with 2,000 linear warm-up steps.

## Results

### Protein function classification

#### Problem setup

Following CLIP [28], our proposed method incorporates a large-scale multi-modal pretraining stage, before executing the cross-modal protein editing. We first conduct experiments to validate that the PLM’s functional protein understanding is enhanced by multi-modality learning with the biotext. Specifically, 4 standard protein function classification benchmarks curated by DeepFRI [51] are employed to classify proteins with multiple functional labels, including Enzyme Commission (EC), Gene Ontology Biological Process (GO-BP), Gene Ontology Molecular Function (GO-MF), and Gene Ontology Cellular Component (GO-CC). Following the previous work [44], we exploit the dataset splits under 95% sequence identity cutoff for both EC and GO. We utilize the multi-modality pretrained PLM for protein function classification. The PLM with pretrained parameter weights extracts the features of protein sequences. Subsequently, a 2-layer parameter randomly initialized MLP (multilayer perceptron) with ReLU nonlinearity in between is introduced to execute function classification by predicting per-function classification logits. Based on such architecture, we perform full fine-tuning for classification tasks, which means that all model parameters are updated, including those of the PLM encoder and MLP prediction head. We consider 4 traditional models (CNN, ResNet, LSTM, and Transformer) and 3 unimodal pretrained PLMs (ProtBERT [20], ESM-1b [25], and ESM-2 [27]) as baselines. Furthermore, we include 2 PLMs (OntoProtein [45] and ProtST-ESM-2 [44]) undergoing multi-modal alignment with the biotext to further evaluate the superiority of our multi-modal pretraining stage. Function classification results are measured by AUPR and $F_{\max}$. AUPR denotes the pair-centric area under the precision–recall curve, and $F_{\max}$ indicates the protein-centric maximum *F* score.

#### Experimental results

As shown in Table 2, ProtET achieves state-of-the-art performance on 6 of 8 evaluation metrics. We observe that ProtET clearly outperforms the vanilla unimodal PLMs and previous multi-modal aligned PLMs, while the large-scale PLMs perform consistently better than traditional models. These results demonstrate that our multi-modal protein–biotext pretraining process is generally beneficial to protein functional understanding, which boosts performance on diverse classification tasks.

**Table 2.** Results of the protein function classification tasks. The best results are in bold and the second-best results are underlined.

Setting	Method	EC		GO-BP		GO-MF		GO-CC	
		AUPR	$F_{\max}$	AUPR	$F_{\max}$	AUPR	$F_{\max}$	AUPR	$F_{\max}$
Traditional models	CNN	0.540	0.545	0.165	0.244	0.380	0.354	0.261	0.387
	ResNet	0.137	0.187	0.166	0.280	0.281	0.267	0.266	0.403
	LSTM	0.032	0.082	0.130	0.248	0.100	0.166	0.150	0.320
	Transformer	0.187	0.219	0.135	0.257	0.172	0.240	0.170	0.380
PLMs under full fine-tuning	ProtBERT	0.859	0.838	0.188	0.279	0.464	0.456	0.234	0.408
	OntoProtein	0.854	0.841	0.284	0.436	0.603	0.631	0.300	0.441
	ESM-1b	0.884	0.869	0.332	0.452	0.630	0.659	0.324	0.477
	ESM-2	0.888	0.874	0.340	0.472	0.643	0.662	0.350	0.472
	ProtST-ESM-2	0.898	0.878	0.342	0.482	0.647	0.668	**0.364**	**0.487**
	ProtET	**0.901**	**0.883**	**0.351**	**0.489**	**0.649**	**0.673**	0.362	0.486

### Enzyme catalytic activity editing

#### Problem setup

**Fig. 4.**
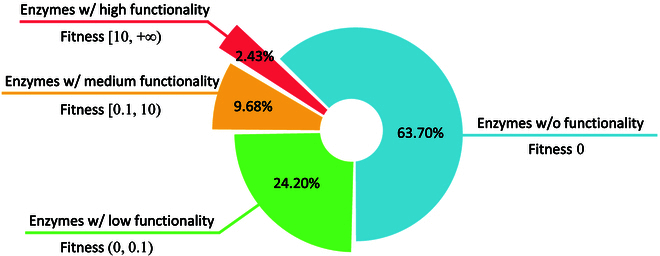
The compositional structure of the enzyme dataset. According to annotated catalytic activity scores, the enzyme dataset is divided into 4 subsets with different fitness levels, and we present the proportion of data for these constructed subsets.

Enzymes are vital proteins that promote metabolism, or the chemical reactions in biological activities. Thus, we conduct a protein editing experiment on publicly available PhoQ [52] dataset, aiming to optimize enzymes toward higher catalytic activity. PhoQ is one of the most widely used dataset to test the protein editing capability of MLPE models. It consists of 140,517 enzymes at 4 sites (A284, V285, S288, and T289), annotated with catalytic activity scores. The catalytic activity scores in PhoQ represent the phosphatase or kinase activity of different PhoQ mutants. Note that we only include the enzymes with thoroughly annotated catalytic activity scores in this task. Given diverse enzymes with annotated catalytic activity scores, we divide the enzyme dataset into subsets with high, medium, and low functionality, as well as a subset without functionality (i.e., enzyme catalytic activity, also described as fitness). The compositional structure of the enzyme dataset with different annotated fitness levels is illustrated in Fig. 4. We respectively sample 100 enzymes from subsets with different fitness levels for the protein editing test and visualization, leaving the remaining proteins for model fine-tuning, and only the aligned key sites are included for loss computation.

#### Experimental results

**Fig. 5.**
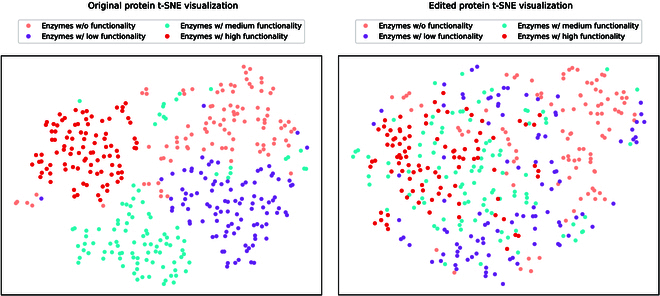
The t-SNE visualization results. Different colors indicate enzymes with different fitness levels correspondingly. Enzymes with medium, low, and zero fitness tend to cluster together with high-functionality enzymes after being edited by ProtET.

We display t-SNE visualization results in Fig. 5. First, we observe that original enzymes with different fitness levels are nicely clustered together, as shown in Fig. 5 (left), indicating that the catalytic activity information can serve as an important clustering basis. Then, we exploit ProtET to perform CLIP-informed protein editing on 3 enzyme subsets with medium, low, and zero fitness, leaving the enzyme subset with high fitness as the editing reference (i.e., enzymes with high fitness serve as the golden standard in this experiment). Owing to the powerful editing capability of ProtET, 3 enzyme subsets initially with poor functionality are optimized and move closer to the editing reference (i.e., enzyme subset with high fitness), as shown in Fig. 5 (right). Such phenomenon manifests that enzymes edited and optimized by ProtET fulfill a remarkable leap in the catalytic activity.

### Protein stability editing

#### Problem setup

To better evaluate the protein editing capability of ProtET, it is worthwhile to conduct convincing experiments. Designing stable proteins is important to ensure, for instance, that drugs are delivered before they are degraded. More generally, given a broad sample of protein measurements, finding better refinements of top stable candidates is useful for maximizing yield from expensive protein engineering experiments. We evaluate ProtET on a set of protein sequences with stability annotations curated by [53]. Starting from original protein sequences, we aim for the edited proteins to exhibit better stability, maintaining its fold above a concentration threshold.

For comprehensive comparison, the stability scores of original protein sequences are included as the bottom line of protein editing performance. We also adopt a single mutation walk approach, referred to as Single-Mutant, as the protein editing baseline [40], which is a hill-climbing algorithm. Single-Mutant [40] performs the single-site mutation across the full length of original protein sequences and selects the mutated protein sequences with the highest stability score as the final edited protein sequences. Furthermore, we compare ProtET with other deep learning-based methods, including AFP-DE [41] and EvoPlay [4]. Concretely, AFP-DE [41] exploits the actively fine-tuned PLM as the sampler and identifies informative mutants that are both representative and diverse. EvoPlay [4] mutates a single-site residue as an action to optimize protein sequences, likening the protein optimization process to playing pieces on a chessboard.

As for the measurement of protein stability, we employ 2 computational approaches to assess the stability scores of edited protein sequences. The simpler one is to directly compute the cosine similarity [49] between the representations of editing instruction texts and edited protein sequences. For the more complex one, we train an MLP according to the experimentally evaluated protein stability labels with a regression loss, and exploit it as the oracle (i.e., surrogate of biological stability experiments) to score stability of edited protein sequences. Further training details of MLP-based oracle are presented in the Supplementary Materials.

#### Experimental results

As shown in Tables 3 and 4, we find that ProtET generates the edited protein sequences with the highest stability under both of the aforementioned assessment criteria. For the first criterion (cosine similarity), ProtET achieves state-of-the-art performance, although other baselines also manifest effective improvement. For the second criterion (oracle), EvoPlay [4] demonstrates considerable improvements over all other baselines, whereas ProtET slightly outperforms EvoPlay.

**Table 3.** Results on the stability score of edited proteins. We record stability scores calculated by cosine similarity here. The best and second-best results are in bold and italic fonts, respectively.

Method	Stability score
Original protein sequences	0.54
Single-Mutant [40]	0.56
AFP-DE [41]	*0.61*
EvoPlay [4]	0.59
ProtET	**0.63**

**Table 4.** Results on the stability score of edited proteins. We record stability scores calculated by the oracle here. The best and second-best results are in bold and italic fonts, respectively.

Method	Stability score
Original protein sequences	0.71
Single-Mutant [40]	0.70
AFP-DE [41]	0.77
EvoPlay [4]	*0.82*
ProtET	**0.83**

**Fig. 6.**
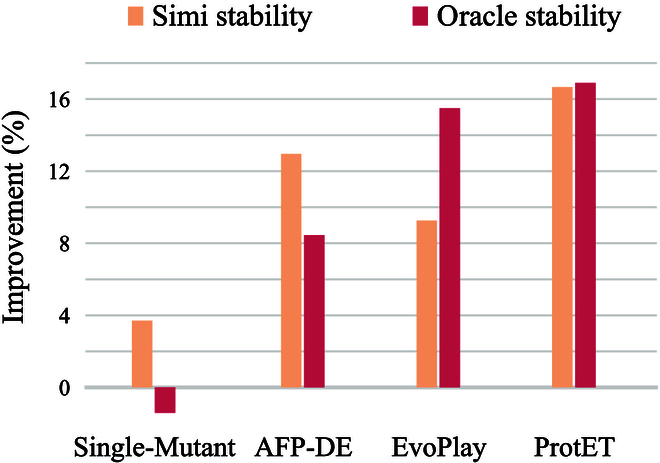
Results on stability improvement of edited proteins. Simi stability: the improvement percentage of stability measured by cosine similarity. Oracle stability: the improvement percentage of stability measured by the conducted oracle.

Furthermore, Fig. 6 displays the stability improvements of edited protein sequences compared to the corresponding original protein sequences. We observe substantial stability enhancements in 16.67% and 16.90% of the proteins edited by ProtET, which adequately illustrates the superiority of the proposed protein editing method.

### Zero-shot SARS-CoV antibody optimization

#### Problem setup

The versatile binding properties of antibodies have made them a substantially important category of proteins [3,54–56]. Among antibodies, complementarity determining regions (CDRs) are the key component to determine the specificity and binding affinity, while CDR-H3 exhibits the highest degree of variability and is hard to predict [57]. Therefore, we further evaluate ProtET to optimize specific binding affinities of SARS-CoV antibodies in a zero-shot manner. We randomly sample 100 antibodies binding to SARS-CoV-1 or SARS-CoV-2 from CoV-AbDab [58]. Note that ProtET focuses exclusively on optimizing CDR-H3 of SARS-CoV antibodies in this task.

Given antibody sequences binding to specific antigens (i.e., SARS-CoV-1 or SARS-CoV-2), we perturb antibody sequences by randomly substituting amino acids in the CDR-H3 region with a 15% probability. Then, ProtET is exploited to edit antibody CDR-H3 fragments with noise, aiming to obtain stronger antigen–antibody binding affinity. Specifically, we employ the pretrained model to generate numerous optimized CDR-H3 fragments, without further fine-tuning on antibody-specific data. For each antibody CDR-H3 fragment with noise, 100 CDR-H3 samples are generated by our ProtET framework. Then, the generated CDR-H3 samples are combined with standard framework regions, enabling 100 full-length generated antibody heavy chains. Since naturalness has been widely proven to be one of the effective indicators reflecting the potential functionality of protein sequences [23,59], we select the top3 antibody heavy chains with the highest naturalness scores using ProGen2 [24].

The optimized antibody sequences are evaluated by whether they possess the capability to fold into regular 3D structures. Concretely, to measure the foldability of the designed antibody sequences, AlphaFold3 [17] and tFold [18] are exploited to predict 3D structures comprising the antigens and designed antibodies (including light chains and selected heavy chains). AlphaFold3 [17] is currently the most authoritative and widely used protein structure prediction tool, accurately predicting the structure of various types of complexes, including the antibody–antigen complex. AlphaFold3 demonstrates substantially improved antibody–antigen prediction accuracy over many previous antibody specialized tools. tFold [18] is the state-of-the-art antibody–antigen complex structure prediction method, and note that it is particularly tailored for antibodies. Additionally, 2 pretrained PLMs (ESM-2 [27] and AntiBERTa [60]), capable of antibody CDR design, are selected as the baseline models for comparison. We perform the structure prediction for designed antibodies, utilizing 3 metrics for structural evaluation: the predicted template-modeling score (pTM), the interface pTM (ipTM), and the predicted local distance difference test (pLDDT).

#### Experimental results

**Fig. 7.**
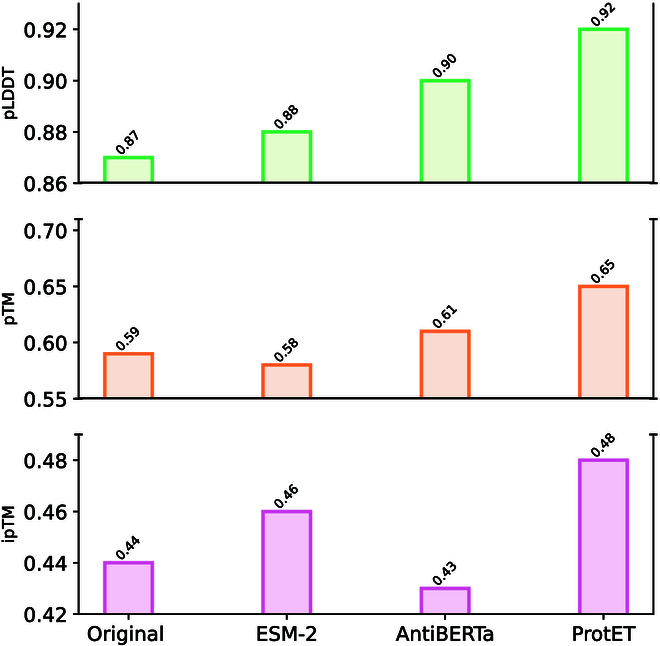
Antibody structural assessments utilizing AlphaFold3. “Original” indicates unedited antibodies with random substitution noise.

**Fig. 8.**
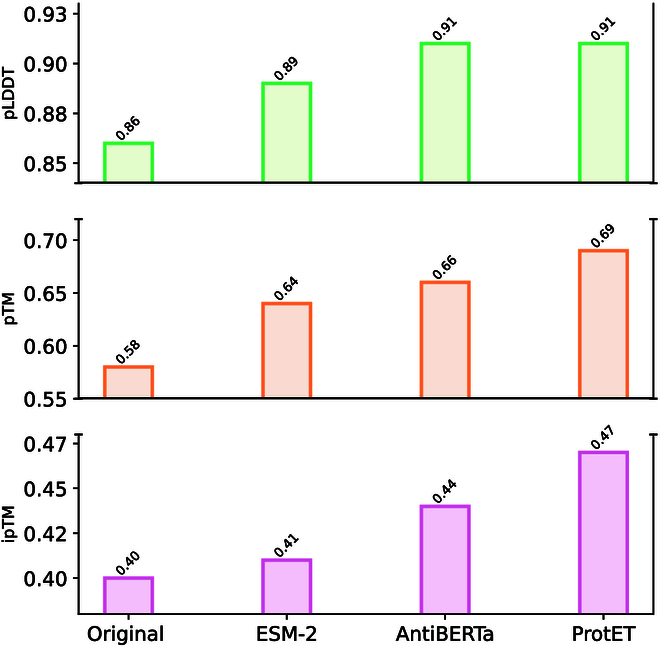
Antibody structural assessments utilizing tFold. “Original” indicates unedited antibodies with random substitution noise.

The structural evaluation results are shown in Figs. 7 and 8. ProtET outperforms other baseline models across all criteria and obtains optimal pTM, ipTM, and pLDDT scores, demonstrating that the edited antibodies are more likely to effectively target their corresponding antigens and form stable binding complexes.

**Fig. 9.**
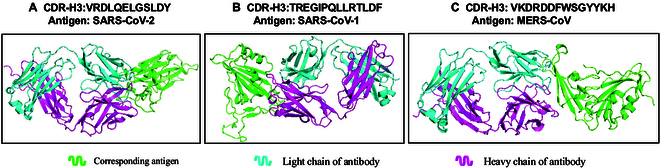
Estimated 3D structures of complexes comprising optimized antibodies and corresponding antigens. (A) The optimized antibody binding to SARS-CoV-2 antigen. (B) The optimized antibody binding to SARS-CoV-1 antigen. (C) The optimized antibody binding to MERS-CoV antigen.

Furthermore, we also provide 3D structural visualization examples designed by ProtET. As illustrated in Fig. 9, optimized antibodies are found to form stable, regular 3D structures that bind with SARS-CoV-1 or SARS-CoV-2 antigens, manifesting that our model successfully designs proteins that satisfy structural constraints.

### Ablation study

In this section, we execute the comprehensive ablation study of ProtET using the protein stability editing dataset described in the “Protein stability editing” section. To thoroughly examine the effectiveness of our proposed innovations, the ablation study focus on following perspectives: (a) The constructed protein–biotext paired dataset uses annotation coverage and evidence-level metrics to filter protein entries from Swiss-Prot and TrEMBL, constructing the high-quality protein–biotext dataset to promote the multi-modality learning. (b) The multi-modal pretraining stage, which is constructed to align the feature spaces of protein and biotext, informed by CLIP [28], lays the groundwork for cross-modal protein editing. (c) The FiLM module is applied for multi-modal feature fusion, with the fused feature acting as the final condition for cross-modal protein editing. We evaluate the essentiality of the FiLM module by replacing it with a simple concatenation operation. As presented in Table 5, we notice that all proposed innovations play an important role in the model’s performance. In particular, the absence of multi-modal pretraining stage yields the highest decrease in performance. The holistic ablation study further validates the superiority of the proposed protein editing method.

**Table 5.** Ablation results of the proposed method on the protein stability editing experiment. CPD denotes the constructed protein–biotext paired dataset, MMP denotes the multi-modal pretraining stage, and FiLM represents the FiLM module for the feature fusion. The best ablation results in this experiment are in bold.

Components			Simi stability	Oracle stability
CPD	MMP	FiLM		
			0.45	0.60
		✓	0.49	0.68
✓	✓		0.60	0.78
✓	✓	✓	**0.63**	**0.83**

## Discussion

Protein molecules are extremely diverse through evolution of 3 billion years [2,61]. Since the space of possible protein molecules is much larger than the space of those likely to have human-expected functions [62], it remains a challenge to accomplish controllable protein discovery and optimization. To alleviate this challenge, we introduce ProtET in this paper, a CLIP-informed protein editing method via protein–biotext multi-modality learning. Compared to existing MLPE models, ProtET proposes a novel hierarchical paradigm for protein editing, encompassing multi-modal pretraining and cross-modal generation. Leveraging the textual functional description as editing instructions, ProtET could be flexibly applied to enhance multiple attributes. This flexibility substantially promotes the practical utility and applicability of our model across different biological contexts. Concretely, we execute multi-modality learning of 2 LLMs to effectively align the feature spaces of the protein and biotext following CLIP [28]. Building upon the multi-modal pretrained encoders, we adopt an autoregressive generative decoder to execute cross-modal protein editing, serving as a pioneering work for artificial intelligence (AI)-assisted controllable protein discovery and optimization. Extensive experiments are constructed to comprehensively assess the performance of the proposed method. Excitingly, given the text editing instruction, the protein sequences generated by ProtET demonstrate excellent functionality, closely aligning with human-expected functional attributes. We select functional attributes from multiple domains, including enzyme catalytic activity, protein stability, and antibody-specific binding ability, to execute controllable protein editing toward human-expected functionality. Revealed by experimental results, ProtET substantially optimizes functional distribution of enzymes (i.e., enzymes initially with poor functionality tend to cluster together with the high-functionality enzymes after being edited by ProtET ). Additionally, ProtET remarkably outperforms other MLPE baseline models (e.g., Single-Mutant [40], AFP-DE [41], and EvoPlay [4]), successfully designing protein sequences with 16.67% and 16.90% stability improvements compared to original protein sequences. Furthermore, optimized antibodies also form stable, regular 3D structures that bind with specific SARS-CoV antigens. If we take a closer look at the proposed method, the core idea of ProtET is to align feature spaces of proteins and biotexts via multi-modality learning, and exploit a cross-modal generation paradigm to accomplish effective and accurate protein editing.

While ProtET bears promise in accelerating AI-assisted controllable protein discovery and optimization, there are still some limitations for improvement and future exploration: (a) Although our model is capable of flexibly editing proteins with desired functional attributes, using natural language as instructions to guide the editing process may entail the issue of insufficient precision, which will be further explored in our future work. (b) When training the proposed protein editing generator, we did not update any parameters of the pretrained large-scale encoders. It may be beneficial to consider incorporating some parameter efficient fine-tuning (PEFT) approaches into pretrained encoders in the future. (c) The autoregressive generation manner falls short in designing proteins with a specified sequence length (i.e., autoregressive generation relies on the probabilistic sampling of the stop token to determine the generated sequence length), leaving some room for exploring more advanced generation paradigm for controllable protein editing. The further exploration will also provide more scientific insights for researchers in real-world applications.

## Conclusion

A major unresolved task in biological research, clinical medicine, and biotechnology is achieving controllable protein discovery and optimization. Protein editing approaches offer a potential solution but still rely heavily on laborious high-throughput screening and experimental heuristics. In this work, we present ProtET, a deep learning-based method for controllable protein editing and optimization. Following CLIP [28], ProtET performs large-scale multi-modal pretraining to align protein and biotext feature spaces and thus well executes the cross-modal protein editing. The target protein we designed exhibited improved functional attributes as expected, and well suited the requirements of multiple domains, including enzyme catalytic activity, protein stability, and antibody-specific binding ability. We hope our work will accelerate the ultimate goal of accomplishing controllable protein discovery and optimization in real-world scenarios.

## Ethical Approval

This study only uses publicly available data, which do not contain sensitive personal information. Therefore, traditional concerns about data privacy and informed consent are not directly applicable. No specific permissions are required for corresponding locations.

## Data Availability

The trained model and source code of this paper are freely available at https://github.com/KDurant-123/ProtET.

## Supplementary Materials

Supplementary 1 Supplementary Text Reference [63]
