# Supplementary material for: Multi-Modal CLIP-Informed Protein Editing
Source: Health Data Sci. 2024 Dec 19;4:0211. doi: 10.34133/hds.0211 (PMC11658819; doi:10.34133/hds.0211)
Supplement: Supplementary 1 — Supplementary Text Reference [63] [file hds.0211.f1.docx]

## **Supplementary Materials**

### 5.1 FiLM module

To optimize protein features based on textual editing instruction features, FiLM module [49] is leveraged to fuse multi-modal features from original protein sequences and editing instruction texts. Specifically, assuming $x$ is a FiLM layer’s input, $z$ is a conditioning input, and __ and __ are *z*-dependent scaling and shifting vectors:

$$FiLM(x)=\gamma(z)\cdot x+\beta(z).$$

We can view the concatenation of all FiLM scaling and shifting coefficients as both an instruction on how to modulate the conditioned network. This process can be understood as incorporating cross-modal conditional information into the original features to attain the fused features. The FiLM layer’s input exists in the original feature space as a dominant entity, while the conditioning input exists in the conditional feature space as a supplementary entity. Therefore, we emphasize that the two input features of the FiLM module cannot be interchanged. In this paper, we utilize the FiLM module to fuse the features of the original protein sequence and the editing instruction text. The resulting fused feature will serve as the final input for auto-regressive generator to design the edited protein. Therefore, we use the original protein sequence feature as the FiLM layer’s input and the editing instruction text feature as the conditioning input, respectively.

### 5.2 Training details of MLP-based oracle

In Section 3.3, we introduce a MLP-based oracle for the rigorous measurement of protein stability, serving as the surrogate of biological stability experiments. The training dataset for this MLP is collected from [64], comprising massive proteins with detailed stability score annotations. These protein stability scores are measured by a novel combination of computational protein design, next-generation gene synthesis, and a high-throughput protease susceptibility assay [64]. The resulting annotated proteins comprehensively cover the landscape of protein stability property. We utilize this holistic dataset for the training of MLP-based oracle, which subsequently serves as the surrogate of wet-lab experiments to assess the protein stability.
